# A survey on the occurrence of *Brachyspira pilosicoli* and *Brachyspira hyodysenteriae *in growing-finishing pigs

**DOI:** 10.12688/f1000research.20639.3

**Published:** 2021-04-01

**Authors:** Arkadiusz Dors, Ewelina Czyżewska-Dors, Grzegorz Woźniakowski

**Affiliations:** 1 Department of Swine Diseases, National Veterinary Research Institute, Puławy, 24-100, Poland

**Keywords:** Brachyspira pilosicoli, Brachyspira hyodysenteriae, Lawsonia intracellularis, pigs, intestinal pathogens, enterocolitis, diarrhea

## Abstract

**Background**: The major pathogenic intestinal spirochetes affecting pigs during the growing- finishing stage of production include *Brachyspira hyodysenteriae* and *Brachyspira pilosicoli*. The aim of this study was to assess the current occurrence of *B. hyodysenteriae* and *B. pilosicoli* in Polish pig herds. Moreover, associations between the presence of diarrhea or other intestinal pathogens and occurrence of *B. hyodysenteriae* and *B. pilosicoli* in pigs were investigated.

**Methods**: Between January 2017 and August 2019, a total of 401 samples of pig feces from 95 different herds were submitted to the National Veterinary Research Institute of Poland. These samples were obtained from pigs older than 7 weeks. All the received fecal samples were examined for the presence of *B. hyodysenteriae*, *B. pilosicoli* and *Lawsonia intracellularis* by real-time PCR.

**Results**: *B. pilosicoli* was detected in 4.5% (95% CI, 2.5–7.0%) (18/401) of pig fecal samples. At the herd level 13.7% (95% CI, 7.5–22.3%) (13/95) of herds were positive for *B. pilosicoli.* *B. hyodysenteriae* was detected in 7.0% (95% CI, 4.7–9.9%) (28/401) of pig fecal samples and 18.9% (95% CI, 11.6–28.3%) (18/95) of pig herds were positive. Out of 18 *B. pilosicoli* positive samples, this pathogen was detected alone in 5 samples; simultaneously with *L. intracellularis *in 9 samples; simultaneously with *B. hyodysenteriae* in 1 sample and in 3 samples was detected simultaneously with both of these bacteria. The presence of *B. hyodysenteriae* in fecal samples was associated with the presence of diarrhea in pigs.

**Conclusions**: This study confirmed that *B. pilosicoli* infections occur in Polish pig herds, but the prevalence is at a low level and the presence of *B. pilosicoli* is not associated with the development of diarrhea in pigs. *B. hyodysenteriae* is still a common cause of diarrhea among pigs from Polish herds.

## Introduction

The question of routine surveillance to monitor *Brachyspira* species infections in pigs at local, national and international levels is addressed by experts and authorities ( Hampson *et al.*, 2015). The major pathogenic intestinal spirochetes affecting pigs during the growing-finishing stage of production include *Brachyspira hyodysenteriae* and *Brachyspira pilosicoli*. *B. hyodysenteriae* is the cause of swine dysentery (SD) – a severe, enteric disease of pigs characterized by mucohemorrhagic diarrhea and inflammation in the large intestine. *B. hyodysenteriae* is present worldwide and affects the economics of pig production, resulting in mortality, growth rate losses and substantial antibiotic costs. Another brachyspiral disease with mild colitis and diarrhea is porcine intestinal spirochetosis or porcine colonic spirochetosis (PIS/PCS). *B. pilosicoli* is the causative agent of PIS/PCS, a disease that implies an important economic cost resulting from reduced growth performance and poor feed conversion ( Duhamel, 1998). Most *Brachyspira* species have a restricted host range, whereas *B. pilosicoli* colonizes a wide range of hosts, including humans, and has the potential for interspecies transmission. There is also a potential for zoonotic transmission, especially in places where animals and humans live in close proximity, or in people working with intensively farmed pigs or chickens, due to the increased risk of exposure. Some species of the genus *Brachyspira*, including *B. pilosicoli*, can cause the disease in humans. There are a few reports on *B. pilosicoli*-associated human intestinal spirochetosis (HIS) ( Hampson, 2018). The subclinical colonization of pigs with *B. pilosicoli* is not uncommon and has been detected in several farms ( Biksi *et al.*, 2007). On other farms, *B. pilosicoli* were isolated from diseased pigs as the only causative agent or simultaneously with other enteric pathogens as part of a mixed infection ( Reiner *et al.*, 2011; Stege *et al.*, 2000).

Recent changes in the management of pig farms and the movement of pigs within the EU have resulted in a shift in the relative prevalence of pathogenic *Brachyspira* species. There are very few studies addressing the prevalence of *B. hyodysenteriae* in pigs in Poland and only one concerning *B. pilosicoli* ( Pławińska *et al.*, 2004). The aim of the study was to assess the current occurrence of *B. hyodysenteriae* and *B. pilosicoli* in Polish pig herds. Moreover, associations between the presence of diarrhea or other intestinal pathogens and the occurrence of *B. hyodysenteriae* and *B. pilosicoli* in pigs were investigated.

## Methods

### Fecal samples

Fecal samples used in this study were submitted to the Department of Swine Diseases of the National Veterinary Research Institute (NVRI) for commercial laboratory diagnostics of selected porcine bacterial pathogens. Between January 2017 and August 2019, a total of 401 samples of pig feces were submitted to the NVRI. These samples originated from 95 different Polish pig herds, from pigs older than 7 weeks. All received fecal samples were submitted to the NVRI to be examined for the presence of *B. hyodysenteriae* and/or *Lawsonia intracellularis*. At that time, none of the diagnostic tools for *B. pilosicoli* identification were available for NVRI customers.

Owing to differing reasons for testing submitted fecal samples, three groups were distinguished. The first group of samples (n=218) were obtained from pigs subjected to routine monitoring of herds free of one or both of the aforementioned pathogens ( *B. hyodysenteriae*, *L. intracellularis*). The second group was made up of samples (n=70) from pigs with clinical signs of diarrhea, where *B. hyodysenteriae* or *L. intracellularis* was suspected to be a cause of disease. The last group of samples (n=113) was submitted to the laboratory due to unrecognized pathogen status and a history of diarrhea in the herd.

### DNA extraction and PCR

Total genomic DNA was extracted from the fecal samples using a commercial isolation kit (Genomic Mini, A&A Biotechnology, Gdynia, Poland), according to manufacturer’s recommendations. Extracted DNA samples were stored at -20°C until examination. All samples were tested by separate singleplex real-time PCR assays for *B. hyodysenteriae* and *L. intracellularis* immediately after samples submission according to the methods described previously ( Zmudzki *et al.*, 2012). Detection of *B. pilosicoli* by real-time PCR was performed in July and August 2019 ( Ståhl *et al.*, 2011). Primers were obtained from a commercial source (Genomed S.A., Poland). The sequences of primers and probes are as follows: for *B. hyodysenteriae* (forward primer: 5'-TATGAAGAAGGCAGCAGACGTTTAT-3', reverse primer: 5'-GTAGGAAGAAGAAATCTGACAATGCA-3', probe: 5'-FAM-ACACAATCATGCTGAAGC-TAMRA-3') ( Akase *et al.*, 2009); for *B. pilosicoli* (forward primer: 5'-GTAGTCGATGGGAAACAGGT-3', reverse primer: 5'-TTACTCACCACAAGTCTCGG-3', probe: 5'-FAM-TATTCGACGAGGATAACCATCACCT-BHQ-1-3') ( Ståhl *et al.*, 2011); for *L. intracellularis* (forward primer: 5'-GCGCGCGTAGGTGGTTATAT-3', reverse primer: 5'-GCCACCCTCTCCGATACTCA-3', probe: 5'-FAM-CACCGCTTAACGGTGGAACAGCCTT-TAMRA-3’) ( Lindecrona *et al.*, 2002). All assays were carried out using the Rotor-Gene Q real-time PCR system (Qiagen, Hilden, Germany).

Real-time PCR assays were run using a commercially available master mix Quantitect Probe PCR kit (Qiagen, Hilden, Germany). For *B. hyodysenteriae*, 12.5 μl of the master mix was combined with 0.5 μl of each primer, diluted to 20 μM and 0.5 μl of the probe, diluted to 20 μM and 6 μl of DNase-free water. The DNA template was added at 5 μl per reaction for a total reaction volume of 25 μl. PCR was run, as follows: 95°C for 15 mins, followed by 50 cycles at 95°C for 15 secs and 52°C for 1 min. For *B. pilosicoli*, 12.5 μl of the master mix was combined with 0.5 μl of each primer, diluted to 20 μM and 0.5 μl of the probe, diluted to 10 μM and 8 μl of DNase-free water. The DNA template was added at 3 μl per reaction for a total reaction volume of 25 μl. PCR was run as follows: 95°C for 15 min, followed by 40 cycles of 95°C for 15 sec and 60°C for 1 min. For *L. intracellularis*, 12.5 μl of the master mix was combined with 0.5 μl of each primer, diluted to 20 μM and 0.5 μl of the probe, diluted to 20 μM and 6 μl of DNase-free water. The DNA template was added at 5 μl per reaction for a total reaction volume of 25 μl. PCR was run as follows: 95°C for 15 min, followed by 40 cycles of 95°C for 15 sec and 62°C for 1 min.

### Statistical analysis

A herd was defined as positive when at least one fecal sample taken from the herd had a positive PCR result. Percentages of positive samples/herds with a 95% two-sides exact binominal confidence interval (CI) were reported. Differences in the presence of pathogens between different fecal samples groups and association with *L. intracellularis* infection were established by a chi-square test (statistically significant at p < 0.05). Pairwise comparisons with Bonferroni corrections of the p-values were performed.

## Results

Among a total of 401 samples 218 were submitted to the NVRI laboratory for the routine monitoring of pig herds. Of these, 70 samples originated from pigs with the clinical manifestation of diarrhea. The remaining 113 samples originated from herds with a history of diarrhea but of an unknown status, in terms of *Brachyspira* spp. occurrence. *Underlying data* are available on figshare ( Dors *et al*., 2019).

*B. pilosicoli* was detected in 4.5% (95% CI, 2.5–7.0%) (18/401) of pig fecal samples. At the herd level 13.7% (95% CI, 7.5–22.3%) (13/95) of herds were positive for *B. pilosicoli. B. hyodysenteriae* was detected in 7.0% (95% CI, 4.7–9.9%) (28/401) of pig fecal samples and 18.9% (95% CI, 11.6–28.3%) (18/95) of pig herds were positive.

Out of 18 *B. pilosicoli* positive samples *,* the pathogen was detected alone in 5 samples; simultaneously with *L. intracellularis* in 9 samples; simultaneously with *B. hyodysenteriae* in 1 sample and in 3 samples was detected simultaneously with both of these bacteria.

Differences in the presence of *B. hyodysenteriae* and *B. pilosicoli* in the fecal samples of various origin (pigs with diarrhea, herds with a history of diarrhea, routine monitoring) are shown in Table 1.

**Table 1.** The differences in the presence of *Brachyspira hyodysenteriae* and *Brachyspira pilosicoli* in fecal samples obtained from healthy pigs and pigs with diarrhea.

Group of fecal samples	Positive samples, %	
	*B. hyodysenteriae*	*B. pilosicoli*
Pig with diarrhea	22.8% *	2.9%
Herds with history of diarrhea	1.8%	1.8%
Routine monitoring	6.3%	6.9%

*Statistically significant difference (p<0.05) between sample groups.

Additional analyses were completed to compare the influence of *L. intracellularis* infection and the presence of *Brachyspira* spp., in fecal samples. The occurrence of *B. hyodysenteriae* in pigs whose feces was confirmed to be positive for *L. intracellularis* was 7.3% (9/123), compared to 6.8% (19/278) in pigs negative for *L. intracellularis* with no statistical significance (p=0.861). However, considering the simultaneous occurrence of *B. pilosicoli* and *L. intracellularis*, we found that the percentage of samples positive for *B. pilosicoli* was significantly higher in pigs simultaneously infected by *L. intracellularis* 9.8% (12/123) compared to *L. intracellularis*-negative pigs 2.2% (6/278) (p<0.001).

## Discussion and conclusions

The results of this study show that *B. pilosicoli* infections occur in Polish pig herds more frequently than it has been thought so far. A previous study reported only one positive sample among 127 samples from 23 pig farms ( Pławińska *et al.*, 2004). Our results show that *B. pilosicoli* is present in Polish pig herds, but that the prevalence is low, reaching 13.7% of herds and 4.5% of samples. Notably, considerably higher prevalence of *B. pilosicoli* infection has been detected in other countries, such as Germany (31.6%, Reiner *et al.*, 2011), Denmark (19%, Stege *et al.*, 2000) and Hungary (61.3%, Biksi *et al.*, 2007). Therefore, the targeted sampling of pigs from age groups in which detection of this pathogen is most likely and random selection of Polish pig herds is necessary to assess the true prevalence of *B. pilosicoli*.

An association between *B. pilosicoli* infections in pigs and the occurrence of diarrhea in this study was not confirmed. Our results are in line with some previous reports ( Biksi *et al.*, 2007; Weber *et al.*, 2015), but other authors have demonstrated positive associations between presence of diarrhea and *B. pilosicoli* detection ( Fellström *et al.*, 1996; Stege *et al.*, 2001). It seems that the subclinical colonization of pigs by *B. pilosicoli* is predominant in pigs, in Poland. Considering the causality of PIS/PCS, other factors causing the development of diarrhea in pigs, besides the *B. pilosicoli* infection, should be considered. *B. pilosicoli* colonization and/or disease expression can be influenced by diet ( Hopwood *et al.*, 2002; Stege *et al.*, 2001). Moreover, concurrent infection can influence *B. pilosicoli* colonization and disease manifestation.

In our study, we have found that samples positive for *L. intracellularis* are more likely to be positive with *B. pilosicoli*. Similar findings have been reported in previous studies ( Biksi *et al.*, 2007; Jacobson *et al.*, 2003; Jacobson *et al.*, 2005; Merialdi *et al.*, 2003). Therefore, there is a need for further investigation to determine a risk factors and an association between the presence of *B. pilosicoli* in feces and the clinical signs or pig performance.

The occurrence of *B. hyodysenteriae* in our investigation was more common than *B. pilosicoli* and was higher than reported previously ( Dors *et al.*, 2015). Current results on the prevalence of *B. hyodysenteriae* could be biased, due to the large number of samples submitted to the NVRI with suspected clinical SD. Nonetheless, SD is still a common cause of diarrhea among pigs from Polish herds, despite improving biosecurity, management and disease control.

In conclusion, our study shows that *B. pilosicoli* infections occur in Polish pig herds, but the prevalence is at a low level and the presence of *B. pilosicoli* is not associated with the development of diarrhea in pigs. Secondly, *B. hyodysenteriae* is still a common cause of diarrhea among pigs from Polish herds. Moreover infection of *L. intracellularis* might be predisposing factor for *B. pilosicoli* occurrence in pigs.

## Data availability

Figshare: A survey on the occurrence of *Brachyspira pilosicoli* and *Brachyspira hyodysenteriae* in growing-finishing pigs. https://doi.org/10.6084/m9.figshare.9878612.v1 ( Dors *et al*., 2019).

This project contains data on detection of infection with each pathogen studied for each sample. 1, yes; 0, no.

Data are available under the terms of the Creative Commons Attribution 4.0 International license (CC-BY 4.0).
