# Supplementary material for: A survey on the occurrence of Brachyspira pilosicoli and Brachyspira hyodysenteriae in growing-finishing pigs
Source: F1000Res. 2021 Apr 1;8:1702. Originally published 2019 Sep 30. [Version 3] doi: 10.12688/f1000research.20639.3 (PMC7993403; doi:10.12688/f1000research.20639.3)
Supplement: Peer review history

# Peer review: Reviewer response for version 2

**DOI:** 10.5256/f1000research.25154.r81218

**Published:** 2021-03-23

**Authors:** Francesca Piras

**Affiliations:** 1 Department of Veterinary Medicine, University of Sassari, Sassari, Italy

The paper “A survey on the occurrence of Brachyspira pilosicoli and Brachyspira hyodysenteriae in growing-finishing pigs” evaluate occurrence of two pathogenic intestinal spirochetes in finishing pigs herd.

 The presented manuscript is well written and sound.

 I have just a few comments:

- **Out of 18 B. pilosicoli positive samples, this pathogen was detected alone in 5 samples.** Change “this” with “the”.
- **The subclinical colonization of pigs, with B. pilosicoli is not uncommon and has been detected in several farms. **The sentence is not clear, delete comma.
- **The second group was made up of samples (n=70) from pigs with clinical sings. **Correct sings with signs.
- **Besides, B. pilosicoli tests were performed in July and August 2019. **The sentence is not clear.

Is the work clearly and accurately presented and does it cite the current literature?

Yes

If applicable, is the statistical analysis and its interpretation appropriate?

Yes

Are all the source data underlying the results available to ensure full reproducibility?

Yes

Is the study design appropriate and is the work technically sound?

Yes

Are the conclusions drawn adequately supported by the results?

Yes

Are sufficient details of methods and analysis provided to allow replication by others?

Yes

Reviewer Expertise:

Microbiology, food safety, enteric pathogens

I confirm that I have read this submission and believe that I have an appropriate level of expertise to confirm that it is of an acceptable scientific standard.

# Response

**Published:** 2021-03-26

**Authors:** arkadiusz dors

I would like to thank the Reviewer for all the comments to our article. I included all the amendments and suggestions in the new version of the manuscript.

# Peer review: Reviewer response for version 2

**DOI:** 10.5256/f1000research.25154.r61312

**Published:** 2020-03-23

**Authors:** Roberto M.C. Guedes

**Affiliations:** 1 Department of Veterinary Clinic and Surgery, Veterinary School, Federal University of Minas Gerais, Belo Horizonte, Brazil

I believe now the manuscript is adequate for indexing!

Is the work clearly and accurately presented and does it cite the current literature?

Yes

If applicable, is the statistical analysis and its interpretation appropriate?

Yes

Are all the source data underlying the results available to ensure full reproducibility?

Yes

Is the study design appropriate and is the work technically sound?

Partly

Are the conclusions drawn adequately supported by the results?

Yes

Are sufficient details of methods and analysis provided to allow replication by others?

Yes

Reviewer Expertise:

NA

I confirm that I have read this submission and believe that I have an appropriate level of expertise to confirm that it is of an acceptable scientific standard.

# Peer review: Reviewer response for version 1

**DOI:** 10.5256/f1000research.22699.r57051

**Published:** 2019-12-05

**Authors:** Roberto M.C. Guedes

**Affiliations:** 1 Department of Veterinary Clinic and Surgery, Veterinary School, Federal University of Minas Gerais, Belo Horizonte, Brazil

**General comments:**

 This study describes the occurrence of pathogenic spirochetes for swine in Poland herds using routine diagnostic cases submitted to their reference laboratory. As a result, sampling was bias for a prevalence study, so the decision of just describe as occurrence was adequate. The data represents important information for Poland swine production, but not so relevant for the rest of the World. Figure 1 is illustrative but it is a repetition of the data that it is already stated in the text. It seems that qPCR for *B. pilosicoli *was not performed at the arrival of the sample, in contrast to qPCR for *B. hyodysenteriae* and *L. intracellularis*. So, when was the qPCR for *B. pilosicoli* performed?

**Specific comments:**

 It was very difficult to list the modification required in the text as it does not have the lines numbered.

 Abstract:

- Results: “…simultaneously with *L. intracellularis. B. hyodysenteriae and B. pilosicoli* were …”

Suggestion of Key-words: pathogenic spirochetes, Lawsonia intracellularis, swine, intestinal pathogens, enterocolitis, diarrhea.

 Results:

- The 4 ^th^ and 5 ^th^ sentences of the second paragraph are confusing. Rewrite.
- The 3 ^rd^ paragraph is too long and confusing. Rewrite.

Is the work clearly and accurately presented and does it cite the current literature?

Yes

If applicable, is the statistical analysis and its interpretation appropriate?

Yes

Are all the source data underlying the results available to ensure full reproducibility?

Yes

Is the study design appropriate and is the work technically sound?

Partly

Are the conclusions drawn adequately supported by the results?

Yes

Are sufficient details of methods and analysis provided to allow replication by others?

Yes

Reviewer Expertise:

enteropathogens of pigs

I confirm that I have read this submission and believe that I have an appropriate level of expertise to confirm that it is of an acceptable scientific standard, however I have significant reservations, as outlined above.

# Response

**Published:** 2020-03-10

**Authors:** arkadiusz dors

I would like to begin by thanking the reviewer for the valuable comments. Below we have included our responses to specific comments.

***Figure 1 is illustrative but it is a repetition of the data that it is already stated in the text.***

Figure 1 was removed because of repetition of results described in the text.

***It seems that qPCR for B. pilosicoli was not performed at the arrival of the sample, in contrast to qPCR for B. hyodysenteriae and L. intracellularis. So, when was the qPCR for B. pilosicoli performed?***

Adequate explanation was added to Methods section.

***Abstract:***

***Results: “…simultaneously with L. intracellularis. B. hyodysenteriae and B. pilosicoli were …”***

Abstract was adjusted to all changes that were made within the article.

***Suggestion of Key-words: pathogenic spirochetes, Lawsonia intracellularis, swine, intestinal pathogens, enterocolitis, diarrhea.***

We found it difficult to change.

***Results:***

***The 4th and 5th sentences of the second paragraph are confusing. Rewrite.***

***The 3rd paragraph is too long and confusing. Rewrite.***

These sentences was rephrased according to reviewer suggestions.

# Peer review: Reviewer response for version 1

**DOI:** 10.5256/f1000research.22699.r55873

**Published:** 2019-11-26

**Authors:** Matheus costa

**Affiliations:** 1 Faculty of Veterinary Medicine, Department of Farm Animal Health, Utrecht University, Utrecht, The Netherlands; 2 University of Minnesota, Minneapolis, USA

I read with great interest this article, that sought to investigate the presence of Bhyo and Bpilo in polish herds. As the authors observed, this is a somewhat challenging goal: both bacterium can survive in the healthy host, thus it's hard to evaluate the meaning of their presence.

 Methods - Fecal samples - Please include a numeric description of each group. You mention the total number of fecal samples, but not how many came from X many herds, and how many are part of each "submission group".

 Results - How many samples were tested out of the 18 that were found positive for Bpilo? Please include the actual numbers in all your descriptions.

 Figure 1 - Please include actual n to the data shown. Also italicize scientific names.

 Lawsonia comparison - Please include actual numbers in all the descriptions (n=?). A statistical test to show that samples positive for LI are more likely to be positive with Bpilo would be interesting here, besides the simple description. 

 Discussion - Was is thought that Poland was free of Bpilo? The first sentence is odd. 

 Please acknowledge that this data set is inherently biased (at least partially, except for the ones that are routine surveillance but we don't know how many samples were part of the group...). 

 Please include a conclusion statement, and clearly lay out your main findings.

Is the work clearly and accurately presented and does it cite the current literature?

Yes

If applicable, is the statistical analysis and its interpretation appropriate?

Partly

Are all the source data underlying the results available to ensure full reproducibility?

Partly

Is the study design appropriate and is the work technically sound?

Yes

Are the conclusions drawn adequately supported by the results?

Partly

Are sufficient details of methods and analysis provided to allow replication by others?

Partly

Reviewer Expertise:

Veterinary medicine, swine medicine, molecular diagnostic tests, microbiome, transcriptome.

I confirm that I have read this submission and believe that I have an appropriate level of expertise to confirm that it is of an acceptable scientific standard, however I have significant reservations, as outlined above.

# Response

**Published:** 2020-03-10

**Authors:** arkadiusz dors

I would like to begin by thanking the reviewer for the valuable comments. Below we have included our responses to specific comments.

***Methods - Fecal samples - Please include a numeric description of each group. You mention the total number of fecal samples, but not how many came from X many herds, and how many are part of each "submission group".***

Number of fecal samples in "submission group" was provided in results section but according to your suggestion we have number also in methods.

***Results - How many samples were tested out of the 18 that were found positive for Bpilo? Please include the actual numbers in all your descriptions.***

Changed as suggested by reviewer.

***Figure 1 - Please include actual n to the data shown. Also italicize scientific names.***

Figure 1 was removed because of repetition of results described in the text.

***Lawsonia comparison - Please include actual numbers in all the descriptions (n=?). A statistical test to show that samples positive for LI are more likely to be positive with Bpilo would be interesting here, besides the simple description.***

Corrected as suggested by reviewer. Necessary explanation has been added to Methods section and p-values were added in the Results.

***Discussion - Was is thought that Poland was free of Bpilo? The first sentence is odd.***

Sentence was rephrased

***Please acknowledge that this data set is inherently biased (at least partially, except for the ones that are routine surveillance but we don't know how many samples were part of the group...).***

In discussion we have mentioned that: *“Current results on the prevalence of B. hyodysenteriae could be biased, due to the large number of samples submitted to the NVRI with suspected clinical SD.”*

***Please include a conclusion statement, and clearly lay out your main findings.***

Final conclusions was added at the end of discussion
